# ^68^Ga-DOTATATE PET/CT for the detection of inflammation of large arteries: correlation with^18^F-FDG, calcium burden and risk factors

**DOI:** 10.1186/2191-219X-2-52

**Published:** 2012-09-27

**Authors:** Xiang Li, Samuel Samnick, Constantin Lapa, Ina Israel, Andreas K Buck, Michael C Kreissl, Wolfgang Bauer

**Affiliations:** 1 Department of Nuclear Medicine, University of Wuerzburg, Oberdürrbacher Str. 6, Wuerzburg, D-97080, Germany; 2 Department of Internal Medicine I, University of Wuerzburg, Wuerzburg, D-97080, Germany; 3 Comprehensive Heart Failure Centre, University Hospital Wuerzburg, Wuerzburg, D-97080, Germany

**Keywords:** Atherosclerotic plaque, ^68^Ga-DOTATATE, Somatostatin receptor, Cardiovascular risk factors, Macrophage

## Abstract

**Background:**

Ga-[1,4,7,10-tetraazacyclododecane-*N*,*N*′,*N*″,*N*′″*-*tetraacetic acid]-d-Phe^1^,Tyr^3^-octreotate (DOTATATE) positron emission tomography (PET) is commonly used for the visualization of somatostatin receptor (SSTR)-positive neuroendocrine tumors. SSTR is also known to be expressed on macrophages, which play a major role in inflammatory processes in the walls of coronary arteries and large vessels. Therefore, imaging SSTR expression has the potential to visualize vulnerable plaques. We assessed ^68^Ga-DOTATATE accumulation in large vessels in comparison to ^18^F-2-fluorodeoxyglucose (FDG) uptake, calcified plaques (CPs), and cardiovascular risk factors.

**Methods:**

Sixteen consecutive patients with neuroendocrine tumors or thyroid cancer underwent both ^68^Ga-DOTATATE and ^18^F-FDG PET/CT for staging or restaging purposes. Detailed clinical data, including common cardiovascular risk factors, were recorded. For a separate assessment, they were divided into a high-risk and a low-risk group. In each patient, we calculated the maximum target-to-background ratio (TBR) of eight arterial segments. The correlation of the TBR_mean_ of both tracers with risk factors including plaque burden was assessed.

**Results:**

The mean TBR of ^68^Ga-DOTATATE in all large arteries correlated significantly with the presence of CPs (*r* = 0.52; *p* < 0.05), hypertension (*r* = 0.60; *p* < 0.05), age (*r* = 0.56; *p* < 0.05), and uptake of ^18^F-FDG (*r* = 0.64; *p* < 0.01). There was one significant correlation between ^18^F-FDG uptake and hypertension (0.58; *p* < 0.05). Out of the 37 sites with the highest focal ^68^Ga-DOTATATE uptake, 16 (43.2%) also had focal ^18^F-FDG uptake. Of 39 sites with the highest ^18^F-FDG uptake, only 11 (28.2%) had a colocalized ^68^Ga-DOTATATE accumulation.

**Conclusions:**

In this series of cancer patients, we found a stronger association of increased ^68^Ga-DOTATATE uptake with known risk factors of cardiovascular disease as compared to ^18^F-FDG, suggesting a potential role for plaque imaging in large arteries. Strikingly, we found that focal uptake of ^68^Ga-DOTATATE and ^18^F-FDG does not colocalize in a significant number of lesions.

## Background

Cardiovascular disease (CVD) has become a global epidemic, being responsible for almost one third of all recorded deaths in men and women worldwide [1,2]. Atherosclerosis is the leading cause of cardiovascular disease morbidity, with the rupture of an atherosclerotic plaque as the critical event resulting in either occlusion of the vessel by a thrombus or thromboembolic event [3].

Cardiovascular inflammatory processes with the accumulation of activated macrophages on the vascular wall play a crucial role in the initiation, progression, destabilization, and eventually, rupture of vulnerable plaques [4]. Therefore, inflammation constitutes an important target for imaging and treating atherosclerosis [5].

There are several invasive methods that can be used to detect atherosclerotic plaques, such as intravascular ultrasonography [6], optical coherence tomography [7], or angiography [8], which is still considered the gold standard. However, due to their invasive nature, they are not recommended for routine clinical application. Numerous noninvasive imaging techniques have been applied to visualize vulnerable plaques, such as MRI and high-frequency ultrasound [9,10]. Recently, other new innovative sensitive imaging methods have been developed [11,12].

Due to its ability to process both structural and functional information, positron emission tomography (PET)/computed tomography (CT) holds great potential in the evaluation of vulnerable plaques. As inflammation detection has become the major concept in atherosclerosis PET/CT imaging, a more sensitive tracer is still to be found. [1,4,7,10-tetraazacyclododecane-*NN*′,*N*″,*N*′″*-*tetraacetic acid]-d-Phe^1^,Tyr^3^-octreotate (DOTATATE) labeled with a generator-derived positron-emitting isotope Gallium-68 (^68^Ga) selectively binds to somatostatin receptor 2 (SSTR-2). ^68^Ga-DOTATATE is routinely used for the staging and restaging of neuroendocrine tumors. Importantly, SSTR-2 was also found specifically expressed and upregulated in human macrophages [13-15], suggesting detection by ^68^Ga-DOTATATE PET imaging. Recently, ^68^Ga-DOTATATE as a more specific alternative to the assessment of macrophages with PET/CT has been published [16]. This tracer may potentially be used as a tool to quantify the extent of atherosclerotic inflammatory activities in big arteries.

Also, ^18^F]-2-fluorodeoxyglucose (FDG), as the most commonly used radiotracer, has already been assessed by several groups [17-19]. Results showed a good correlation between carotid plaque ^18^F-FDG uptake *in vivo* and macrophage staining from the corresponding histological sections [20,21]. Furthermore, there was a weak but highly significant correlation between ^18^F-FDG uptake and cardiovascular risk factors like hypertension, smoking, hyperlipidemia, being overweight, type II diabetes, and a family history of coronary artery disease (CAD) [22].

The purpose of this retrospective study was to examine the relationship of focal vascular ^68^Ga-DOTATATE uptake to commonly known risk factors for cardiovascular disease and calcification. We also performed a correlation to vascular ^18^F-FDG uptake, which has been demonstrated to be a marker for atherosclerotic inflammation in large arteries [23-26].

## Methods

### Patients

A total of 16 patients (12 males and 4 females, age ranges from 48 to 77 years, mean age was 63.5 years) were retrospectively reviewed by two experienced nuclear medicine physicians (MCK and CL). These patients had undergone both ^68^Ga-DOTATATE PET/CT and ^18^F-FDG PET/CT for staging or restaging within 6 weeks (mean, 3.8 weeks; range, 0.14 to 5.9). None of these patients received steroids or had a recent history of inflammation or vasculitis. The study protocol complied with the Declaration of Helsinki; all patients gave their informed consent for the studies.

### Imaging procedures

All patients underwent ^18^F-FDG PET/CT and ^68^Ga-DOTATATE PET/CT on a dedicated PET/CT scanner (Siemens Biograph ® mCT 64, Siemens, Knoxville, USA) consisting of a LSO full-ring PET and a 64-slice spiral CT. On the day of the ^18^F-FDG PET imaging, patients fasted for at least 6 h to assure a serum glucose level below 130 mg/dL. At the time point of intravenous injection of 4.18 ± 0.88 MBq/kg bodyweight, patients received 10 mg of furosemide i.v. After an uptake period of 80 to 90 min, transmission data were acquired using a low-dose CT (30 mAs, 120 kV, a 512 × 512 matrix, a 5-mm slice thickness, an increment of 30 mm/s, a rotation time of 0.5 s, and a pitch index of 0.8) extending from the base of the skull, or the vertex, to the proximal thighs. Consecutively, PET emission data were acquired in three-dimensional mode with a 200 × 200 matrix with 2-min emission time per bed position. After decay and scatter correction, PET data were reconstructed iteratively with attenuation correction using a dedicated software (HD. PET, Siemens Esoft).

For the assessment of somatostatin receptor expression, a mean dose of 1.29 ± 0.43 MBq ^68^Ga-DOTATATE/kg bodyweight was injected intravenously. After a period of 40 to 60 min, CT and PET data acquisition was started using the same parameters as mentioned above.

### Image analysis

All PET/CT scans were reviewed for anatomic localization and amount of focal tracer uptake. In each patient, eight segments of large arteries were analyzed: left carotid artery, right carotid artery, ascending thoracic aorta, aortic arch, descending thoracic aorta, abdominal aorta, and left and right iliac arteries.

Arterial calcifications were also assessed; vascular attenuation of >130 Hounsfield units was rated as calcification [27,28]. The plaque burden was semiquantitatively determined by assessing the maximal dimensions and composition of plaque using a scoring system from 0 to 4, as previously described by Rominger et al. [16] (Table 1).

**Table 1:** Calcified plaque scoring system

**Score**	**Calcified occupation of the vessel circumference (%)**
0	0
1	<10
2	10 to 25
3	25 to 50
4	>50

The total calcified plaque burden for each of the eight arterial segments was defined as the sum of the individual calcified plaque scores.

For PET data analysis, a region of interest (ROI)-based approach was chosen. Maximal ‘standardized uptake values’ (SUV_max_) for both ^18^F-FDG and ^68^Ga-DOTATATE uptake were calculated for all the segments mentioned above [29]. Areas in proximity to tumor lesions or organs with a high physiological tracer uptake, i.e., the liver, were carefully avoided. At least three fixed-size ROIs were placed to cover the lumen at each site, and the highest value was extracted for the final calculation of target-to-background ratios (TBRs). Background was defined as the average blood-pool uptake as determined by the mean SUV of six different ROIs (diameter of 1 cm) within the lumen of the vena cava. The SUV_max_ was divided by the mean blood-pool SUV in order to obtain the TBR [22]. We used the TBRs of all eight arterial segments for the final analysis. To determine whether sites of increased focal uptake of ^18^F-FDG and ^68^Ga-DOTATATE colocalize, the sites with the highest uptake (within about 30% of the highest uptake value) were assessed. Accordingly, arbitrary cutoff TBR values of 3.5 for ^68^Ga-DOTATATE and 2.1 for ^18^F-FDG were chosen, resulting in 37 foci of increased ^68^Ga-DOTATATE uptake and 39 foci of increased FDG uptake. Each of these foci was visually assessed for the presence of concordantly increased focal uptake of the other tracer and for the presence of calcifications in the vessel wall.

We also assessed the differential vascular uptake of the two tracers in a low-risk group for CAD, defined as subjects with no more than one cardiovascular risk factor (*n* = 8), and a high-risk group with at least two cardiovascular risk factors (*n* = 8). For ROI assignment, visualization of PET/CT images, and automated coregistration of the two PET/CT datasets, a dedicated software (TrueD, Siemens Healthcare) was used.

### Statistical methods

Statistical Package for Social Sciences (SPSS version 11.0; SPSS Inc. (IBM), Armonk, NY, US) was used for statistical analyses. Continuous variables with a normal distribution were recorded as mean ± standard deviation. The site-specific blood-pool activities of the two tracers were compared using ANOVA. Pearson correlation coefficients were used for the assessment association between the uptake of the two tracers and the presented cardiovascular risk factors: plaque burden, hypercholesterolemia, hypertension, smoking, diabetes, family history, history of cardiovascular disease, body mass index (BMI), age, and gender. Moreover, intraclass correlation coefficients (ICCs) with 95% confidence intervals were calculated to test interobserver and intraobserver agreement for TBR. Two-way random ICC values that are greater than 0.8 are accepted as a measure of excellent reproducibility [30].

## Results

### Patient population

Relevant baseline characteristics of the patients are reported in Table 2. In brief, 8 out of 16 patients had a history of neuroendocrine tumors, three suffered from thyroid cancer, and the remaining five patients had different carcinomas. Systemic tumor therapy preceding imaging for less than 12 weeks was administered in four patients. Detailed information on tumor types and therapies can be found in Table 3, and cardiovascular risk factors can be found in Table S6 in Additional file 1.

**Table 2:** Patient characteristics

**Characteristics**	
Age (yrs)	63.5 ± 8.8
Gender (M, F)	4, 12
Hypercholesterolemia (%)	31.25
Hypertension (%)	56.25
Smoking (%)	25
Diabetes mellitus (%)	12.5
Family history of CAD (%)	25
History of CVD (%)	18.75
CPB	9.44 ± 8.07

CAD, coronary artery disease; CVD, cardiovascular disease; CPB, calcified plaque burden; F, female; M, male.

**Table 3:** Baseline characteristics of the study population

**Patient number**	**Cancer type**	**Systemic tumor therapy within the last 3 months**	**Number of selected positive-uptake segments**		**TBR**_**mean**_	
			**FDG**	**DOTATATE**	**FDG**	**DOTATATE**
1	Renal cell cancer	None	8	6	2.9	5.6
2	Follicular thyroid cancer	TSH-suppressive therapy	7	2	2.6	3.3
3	Pheochromocytoma, metabolically active (normetanephrines, dopamines)	None	3	4	2.1	3.9
4	Cancer of the gastric-esophageal junction	Xeloda, single dose 2 days prior to FDG, 6 days prior to DOTATATE PET	0	0	1.5	1.8
5	NET (stomach)	None	0	0	1.7	2.2
6	NET of unknown primary (liver metastases)	None	6	3	2.2	3.4
7	Carcinoid (rectum)	One cycle of Lu-177-DOTATATE 8 weeks prior to FDG, 9 weeks to DOTATATE PET	0	1	1.7	2.9
8	NET of unknown primary (liver metastases)	None	5	2	2.1	2.9
9	NET (pancreas)	One cycle of cisplatin/etoposide 1 week after FDG; 4 weeks prior to DOTATATE PET)	2	1	1.8	2.4
10	Carcinoid (lung), hormonally active	None	1	2	1.7	2.6
11	NET of unknown primary (liver metastases)	Sandostatin LAR, last injection 4 weeks prior to DOTATATE PET, then stopped	0	0	1.5	2
12	NET (ileum)	None	2	1	1.7	2.8
13	NSCLC	None	1	8	1.8	5.7
14	Papillary and follicular thyroid cancer	TSH-suppressive therapy	2	4	1.9	3.5
15	Follicular thyroid cancer	TSH-suppressive therapy	2	3	1.9	3.1
16	Uterine cancer	None	0	0	1.4	1.3

NET, neuroendocrine tumor; NSCLC, non-small cell lung cancer; TSH, thyroid-stimulating hormone.

### PET/CT imaging

**Figure 1:**
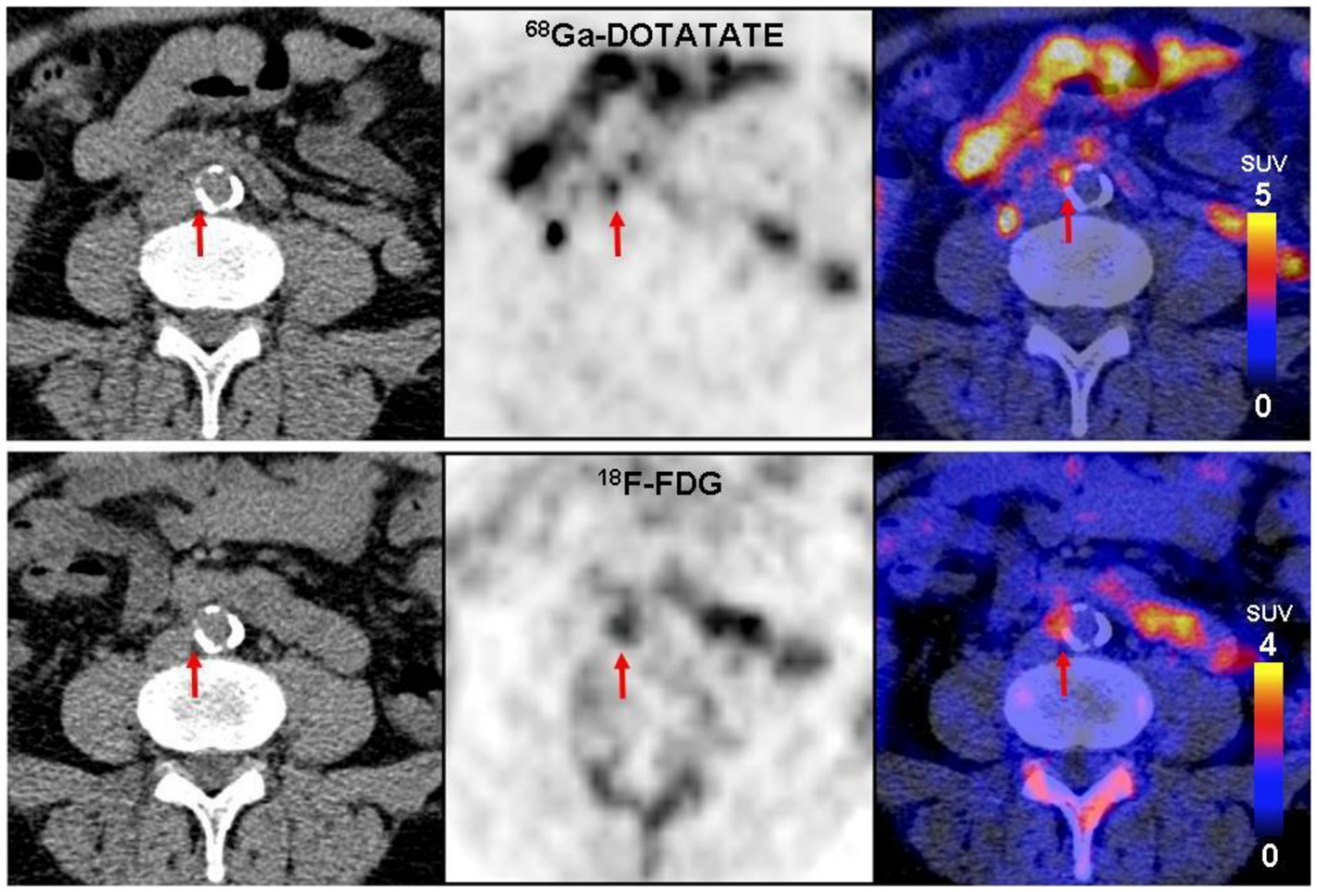
**Colocalized focal vascular uptake of **^**18**^**F-FDG and **^**68**^**Ga-DOTATATE.** Transverse views of a 61-year-old male patient with hypertension and a history of cardiovascular disease. Positive uptake is present on both ^68^Ga-DOTATATE (upper row) and ^18^F-FDG PET/CT (lower row) at the same location in the abdominal aorta of this patient (red circle). Also, serious calcification was detected at the same position. TBR_DOTATATE_ was 6.18, while TRB_FDG_ was 2.42.

**Figure 2:**
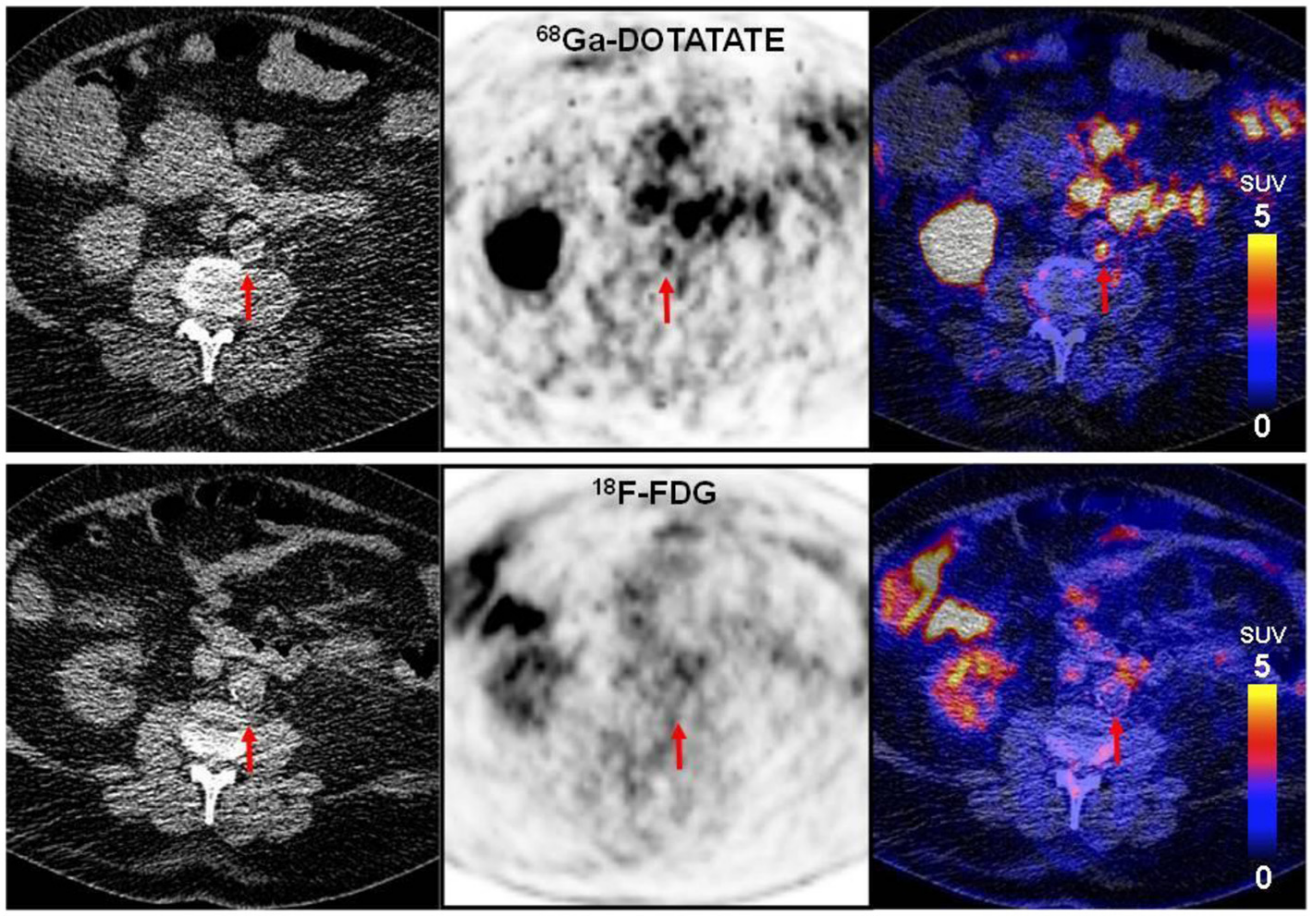
**Focal vascular uptake of **^**68**^**Ga-DOTATATE without corresponding focal **^**18**^**F-FDG uptake.** Transverse PET/CT images of a 73-year-old male patient with hypertension, hypercholesterolemia, and smoking. Intense focal uptake of ^68^Ga-DOTATATE can be observed in the aortic arch (upper row), whereas no focally increased ^18^F-FDG uptake was seen (lower row). TBR_DOTATATE_ was 7.60, while TRB_FDG_ was 1.74.

**Figure 3:**
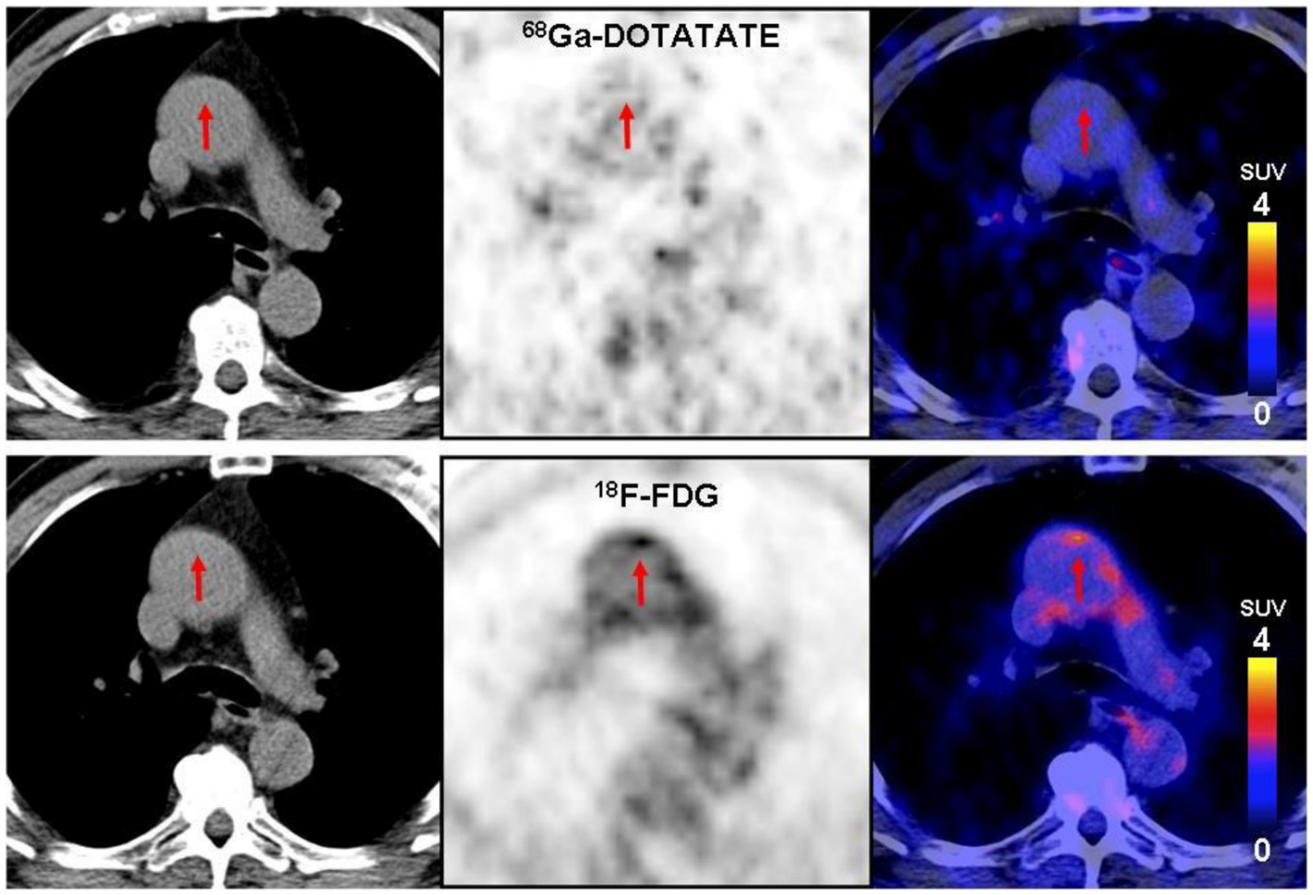
**Focal uptake of FDG but not DOTATATE in the vessel wall.** Transverse PET/CT images of a 77-year-old male patient. With hypertension, hypercholesterolemia, history of coronary disease and a family history of cardiovascular disease. Intense ^18^F-FDG uptake can be observed in the aortic arch (upper row), whereas no focally increased ^68^Ga-DOTATATE uptake can be seen (lower row). TBR_DOTATATE_ was 1.64, while TRB_FDG_ was 2.50.

**Figure 4:**
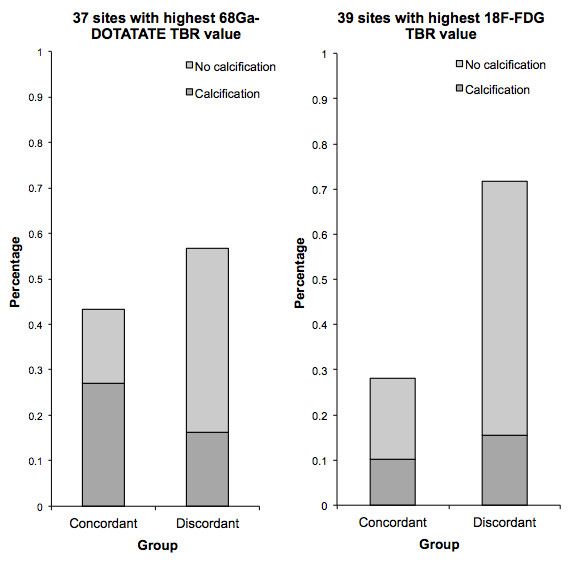
**Colocalization of focal **^**68**^**Ga-DOTATATE and **^**18**^**F-FDG uptake.** Comparison of 37 foci of increased ^68^Ga-DOTATATE uptake (left) and 39 foci of increased ^18^F-FDG uptake (right) with respect to colocalized focal uptake of the other radiotracer and calcification. Imaging findings were characterized as concordant when there was agreement in positive detection with both tracers, and as discordant in cases of discrepancy between the two tracers' uptake.

When assessing the PET/CT image data, we found colocalized uptake of both tracers (Figure 1) as well as foci of increased uptake of only one of the tracers (Figures 2 and 3). On evaluation of the 37 sites with the highest ^68^Ga-DOTATATE TBR value, a colocalized focal increase of ^18^F-FDG uptake was observed in 43.2% (16 sites, 10 sites with calcification, and 6 sites without calcification), while 56.8% of the sites (21 sites, 6 sites with calcification, and 15 sites without calcification) were negative. Of 39 sites with the highest ^18^F-FDG uptake, only 28.2% had an increased uptake of ^68^Ga-DOTATATE (11 sites, 4 sites with calcification, and 7 sites without calcification), while 71.8% of the sites (28 sites, 6 sites with calcification, and 22 sites without calcification) were recorded as negative (Figure 4). In total, 12 patients were examined to have focal increased uptakes of ^68^Ga-DOTATATE, while 10 patients were found to have ^18^F-FDG focal increased uptakes. As previously recommended, we also used the mean TBR, which was calculated as the mean value of TBRs from eight arterial segments, for statistical analyses.

### Subgroup analysis

**Figure 5:**
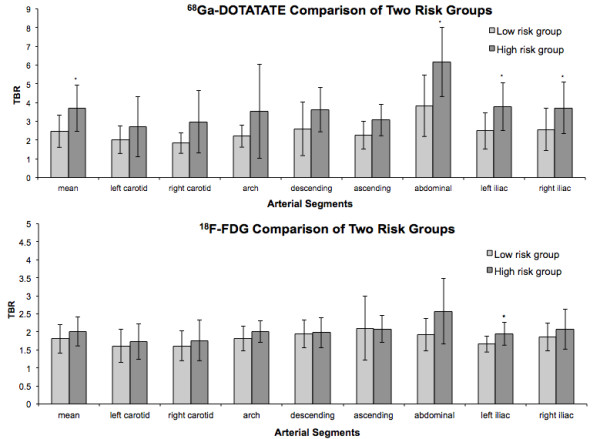
**Comparison between high- and low-risk groups for cardiovascular disease.** With regard to the ^18^F-FDG and ^68^Ga-DOTATATE uptake of big arterial vessels and calcified plaques. One asterisk indicates that comparison is significant at the 0.05 level (two-tailed).

Two risk-related groups were formed to assess the differential vascular uptake of the two tracers. The summed calcification scores (CP_sum_) differed significantly between the two groups (*p* < 0.05). The TBR_mean_, TBR_abdominal_, TBR_left_iliac_, and TBR_right_iliac_ of ^68^Ga-DOTATATE were significantly higher in the high-risk group (at least two cardiovascular risk factors) as compared to the low-risk group (one cardiovascular risk factor at the most) (Figure 5). In contrast, for ^18^F-FDG, the TBR_mean_ and TBR_abdominal_ only showed a tendency for a higher uptake in the high-risk group; only the TBR value in the left iliac artery was found to be significantly higher (*p* < 0.05).

### Correlation with risk factors

Table 4 shows the correlation of ^18^F-FDG and ^68^Ga-DOTATATE TBR_mean_ values with clinical baseline characteristics. Hypertension significantly correlated with ^18^F-FDG (*r* = 0.58, *p* < 0.05) and also with ^68^Ga-DOTATATE uptake (*r* = 0.60, *p* < 0.01). The ^68^Ga-DOTATATE TBR_mean_ also showed a significant correlation with plaque burden (*r* = 0.52, *p* < 0.05), hypertension (*r* = 0.60, *p* < 0.05), and age (*r* = 0.56, *p* < 0.05). In addition, plaque burden was also significantly correlated with smoking (*r* = 0.55, *p* < 0.05) and a history of cardiovascular disease (*r* = 0.63, *p* < 0.01). There was a significant correlation between the two tracers' uptake (*r* = 0.64, *p* < 0.05).

**Table 4:** **Correlation of **^18^**F-FDG TBR**_**mean **_**and **^68^**Ga-DOTATATE TBR**_**mean **_**of eight arterial segments to the baseline characteristics**

**Pearson's correlation coefficients for the correlation between risk factors and imaging results**												
	**Mean**^**18**^**F-FDG**	**Mean**^**68**^**Ga-DOTATATE**	**PB**	**Hyperchol**	**HTN**	**Smoker**	**DM**	**Family history of CVD**	**CVD**	**Age**	**Gender**	**BMI**
Mean ^18^F-FDG	1	0.64^a^	NS	NS	0.58^b^	NS	NS	NS	NS	NS	NS	NS
Mean ^68^Ga-DOTATE	0.64^a^	1	0.52^b^	NS	0.60^b^	NS	NS	NS	NS	0.56^b^	NS	NS
PB	NS	0.52^b^	1	NS	NS	0.55^b^	NS	NS	0.63^a^	NS	NS	NS

^a^Correlation is significant at the 0.01 level (two-tailed). ^b^Correlation is significant at the 0.05 level (two-tailed). BMI, body mass index; CAD, coronary artery disease; CVD, cardiovascular disease; DM, diabetes; HTN, hypertension; Hyperchol, hypercholesterolemia; NS, no significance; PB, plaque burden.

### Reproducibility

The intraclass correlation coefficient values with 95% confidence were calculated for the maximum TBR and CPB of all arteries and are shown in Table S3 in Additional file 1. All ICCs were greater than 0.8.

## Discussion

Our study was aimed at investigating the potential of ^68^Ga-DOTATATE PET/CT to detect macrophage density at inflammatory lesions of large arteries. In addition, we compared the tracer uptake with that of ^18^F-FDG regarding colocalization and intensity of the uptake in correlation to commonly accepted risk factors for cardiovascular disease. To our knowledge, this is the first investigation to compare ^18^F-FDG and ^68^Ga-DOTATATE in atherosclerosis imaging.

^18^F-FDG has already been established as a useful tool to identify inflammatory processes, e.g., in large arteries [17-22]. In general, it is taken up by cells in proportion to their metabolic activity. Rudd et al. and Tawakol et al. reported that carotid atherosclerotic plaques with high ^18^F-FDG uptake have a high macrophage density [20,21], indicating that ^18^F-FDG might be a useful tool to detect inflammatory plaques. ^68^Ga-DOTATATE has also been demonstrated to detect activated macrophages [16] due to a specific overexpression of the SSTR-2, the specific molecular target of ^68^Ga-DOTATATE, on the cell surface of activated macrophages. Although the potential for macrophage detection has been demonstrated on both ^18^F-FDG and ^68^Ga-DOTATATE, a concordant focally increased uptake was only found in the minority of cases in this study (Figure 4).

When comparing patients with and without risk factors for cardiovascular disease, many sites of tracer uptake could be detected both in high-risk and low-risk patients with ^18^F-FDG. In most arterial segments (apart from the ascending aorta), the maximum TBR values of the high-risk group were higher than those of the low-risk group. However, this difference did not reach statistical significance except for the left iliac artery (Figure 5).

There were few significant differences between the two tracers in the low-risk group. In the high-risk group, however, TBR for ^68^Ga-DOTATATE showed significantly higher values than that for ^18^F-FDG.

Additionally, significant correlations between the mean uptake of both tracers and the patients' score of risk factors were found. In the literature, several studies reported significant correlations between focally increased ^18^F-FDG uptake in the walls of large arteries and the presence of cardiovascular risk factors, such as hypercholesterolemia, hypertension, age, and diabetes [24]. Other study groups found associations between the plaque burden of the left anterior descending coronary artery and cardiovascular risk factors [22,31]. In our study, only a significant correlation between the mean TBR in the abdominal aorta with hypertension was found. The lack of further correlations might be due to the small size of our sample. For ^68^Ga-DOTATATE, tracer uptake correlated significantly with hypertension, age, and the presence of calcifications.

^18^F-FDG uptake in inflammatory cells is influenced by macrophage differentiation and cell activation [25] since immune cell activation is associated with increased oxidative metabolism and, consequently, increased use of glucose [18]. In our investigation, we found many ^18^F-FDG-positive sites both in the low- and high-risk groups, whereas very few ^68^Ga-DOTATATE-positive foci could be found in low-risk individuals. This raises doubt whether all accumulations of ^18^F-FDG within the arteries are due to macrophage-mediated inflammatory activity.

In contrast to ^18^F-FDG, ^68^Ga-DOTATATE visualizes the distribution of SSTR-2. As mentioned above, the specific expression of SSTR-2 on the surface of macrophages, as well as the significant upregulation of this receptor upon stimulation (e.g. with lipopolysaccharide), has been reported [14]. Furthermore, another research group observed that the expression of SSTR-2 in human coronary endothelial cells is decreased by treatment with the inflammatory cytokine TNF-α, which is mainly produced and secreted by activated macrophages [26]. Therefore, in the pro-inflammatory setting, SSTR-2 expression is upregulated for macrophages, whereas on the other hand, it is downregulated in endothelial cells. In coronary heart disease, this idea could support the use of ^68^Ga-DOTATATE for the detection of vulnerable plaques.

SSTR-2 also plays another role in atherosclerotic plaques. Adams et al. demonstrated the increased expression of SSTR-2 when human umbilical vein endothelial cells are proliferating, thereby suggesting its active role in angiogenesis, which has been described in the context of unstable plaques [32]. Consequently, ^68^Ga-DOTATATE PET could be used to visualize unstable plaques in two different ways: detection of activated macrophages as well as angiogenesis within the atherosclerotic lesion [33]. Our results indicate that ^68^Ga-DOTATATE PET detects a higher number of increased focal uptakes in patients with high cardiovascular risk; this may be used as a complementary imaging modality in the detection of inflammatory plaque by identifying somatostatin receptor-positive sites within the arteries.

In this study, risk factors for CAD correlated less strongly with foci of increased ^18^F-FDG uptake as compared to those of ^68^Ga-DOTATATE. This might be interpreted as an indication that ^68^Ga-DOTATATE is more specific for the inflamed plaque. However, due to the small sample size of our investigation, it is difficult to draw firm conclusions. For example, another reason for the observed difference between the two tracers could be tracer kinetics*.* DOTATATE binds to the SSTR-2, and thus, the uptake is potentially limited by the number of saturated SSTR-2 receptors. This is not the case with FDG, which is continuously metabolized by activated macrophages. Otherwise, FDG as the smaller molecule is potentially more vulnerable to unspecific uptake mechanisms like increased vascular permeability due to inflammation. So, in theory, FDG should be more sensitive than DOTATATE but potentially less specific, which could explain the better correlation with different risk groups.

Another important limitation is the lack of histological validation of ^68^Ga-DOTATATE uptake. Due to the retrospective design, a selection bias cannot be excluded. Also, this study was performed in cancer patients; therefore, our findings may be not generalizable to studies in vascular disease. Especially, we cannot exclude the influence of anticancer therapies or the hormone action of neuroendocrine tumors (Table 3). So far, no influence of TSH-suppressive therapy or peptide receptor radionuclide therapy (PRRT) on atherosclerosis has been published. In the only patient with PRRT, the interval to the PET scans was at least 8 weeks, so we do not expect any interference. Also, none of the two patients on chemotherapy was immunodeficient, according to their white blood cell count. However, further prospective studies in a higher number of non-oncological patients are warranted.

## Conclusions

In this pilot trial, we found a stronger association of increased ^68^Ga-DOTATATE uptake with known risk factors of cardiovascular disease as compared to ^18^F-FDG in a limited number of oncological patients, suggesting a potential role of this tracer for plaque imaging in the large arteries and coronaries. Strikingly, we found that the focal uptake of ^68^Ga-DOTATATE and ^18^F-FDG does not colocalize in a significant percentage of lesions. This warrants further investigation on the distribution of the two tracers within atherosclerotic plaques.

## Competing interests

The authors declare that they have no competing interests.

## Authors' contributions

XL conducted the study, analyzed the data, and wrote the manuscript. SS supervised the work and gave input to the manuscript. CL analyzed the image data as well as clinical data and corrected the manuscript. II synthesized the radiotracer. AKB supervised the study and the assessments and directed the focus of the research. MCK conceived of the study and study layout, performed the image analysis, and corrected and revised the manuscript. WB supervised the research area. All authors read and approved the final manuscript.

## Supplementary Material

Additional file 1 This file contains supplemental Tables S1, S2, S3, S4, S5, and S6. Click here for file
