# Supplementary material for: 68Ga-DOTATATE PET/CT for the detection of inflammation of large arteries: correlation with18F-FDG, calcium burden and risk factors
Source: EJNMMI Res. 2012 Sep 27;2:52. doi: 10.1186/2191-219X-2-52 (PMC3475087; doi:10.1186/2191-219X-2-52)
Supplement: Additional file 1 — This file contains supplemental Tables S1, S2, S3, S4, S5, and S6. [file 2191-219X-2-52-S1.doc]

**Supplemental Files**

**TABLE 1:**

Comparison between high and low-risk group for cardiovascular disease, in regard to the ^18^F-FDG and ^68^Ga-DOTATATE uptake of big arterial vessels and calcified plaques

| Parameters |  |  | Risk Group |  |  |
| --- | --- | --- | --- | --- | --- |
|  |  |  | Low Risk (n=8)  (95% CI for mean) | High Risk (n=8)  (95% CI for mean) | Sig.  (p value) |
| No. of risk factors |  |  | 0.43 | 2.88 |  |
| Age |  |  | 59±8.21 (48;71) | 67±8.42 (52;77) |  |
| Mean (Range) | ^18^F-FDG | TBR_mean_ | 1.81±0.40 (1.48;2.14) | 2.01±0.40 (1.68;2.35) | 0.315 |
|  | ^68^Ga-DOTATATE | TBR_mean_ | 2.47±0.86 (1.76;3.19) | 3.70±1.24 (2.66;4.74) | 0.038 |
|  | CP sum |  | 4.75±5.23 (0.38;9.12) | 14.12±7.86 (7.55;20.7) | 0.014 |
| TBR (Range) | ^18^F-FDG | TBR_left carotid_ | 1.61±0.46 (1.22;1.99) | 1.73±0.49 (1.31;2.14) | 0.626 |
|  |  | TBR_right carotid_ | 1.61±0.41 (1.27;1.96) | 1.76±0.56 (1.29;2.22) | 0.565 |
|  |  | TBR_arch_ | 1.81±0.34 (1.52;2.10) | 2.01±0.30 (1.76;2.27) | 0.227 |
|  |  | TBR_descending_ | 1.94±0.39 (1.62;2.27) | 1.98±0.42 (1.64;2.33) | 0.835 |
|  |  | TBR_ascending_ | 2.10±0.89 (1.36;2.85) | 2.08±0.38 (1.76;2.40) | 0.948 |
|  |  | TBR_abdominal_ | 1.92±0.45 (1.54;2.29) | 2.57±0.91 (1.81;3.32) | 0.092 |
|  |  | TBRleft_iliac | 1.66±0.22 (1.43;1.81) | 1.95±0.32 (1.68;2.21) | 0.03 |
|  |  | TBRright_iliac | 1.86±0.39 (1.53;2.19) | 2.07±0.55 (1.61;2.52) | 0.403 |
|  | ^68^Ga-DOTATATE | TBR_left carotid_ | 2.02±0.74 (1.41;2.64) | 2.71±1.60 (1.37;4.05) | 0.29 |
|  |  | TBR_right carotid_ | 1.84±0.55 (1.38;2.30) | 2.97±1.67 (1.57;4.38) | 0.089 |
|  |  | TBR_arch_ | 2.20±0.58 (1.71;2.68) | 3.53±2.51 (1.44;5.63) | 0.164 |
|  |  | TBR_descending_ | 2.60±1.43 (1.40;3.80) | 3.63±1.19 (2.63;4.63) | 0.139 |
|  |  | TBR_ascending_ | 2.26±0.74 (1.64;2.87) | 3.07±0.84 (2.38;3.77) | 0.057 |
|  |  | TBR_abdominal_ | 3.82±1.63 (2.45;5.18) | 6.17±1.85 (4.62;7.71) | 0.017 |
|  |  | TBRleft_iliac | 2.49±0.98 (1.67;3.31) | 3.78±1.29 (2.70;4.86) | 0.041 |
|  |  | TBRright_iliac | 2.56±1.13 (1.62;3.50) | 3.71±1.37 (2.56;4.86) | 0.048 |

**TABLE 2.**

Comparison between ^18^F-FDG and ^68^Ga-DOTATATE uptake of big arterial vessels within high and low-risk group respectively

|  |  | ^18^F-FDG  (95% CI for mean) | ^68^Ga-DOTATATE (95% CI for mean) | Sig. (p value) |
| --- | --- | --- | --- | --- |
| Low Risk (n=8) | TBR_mean_ | 1.81±0.40 (1.48;2.14) | 2.47±0.86 (1.76;3.19) | 0.067 |
|  | TBR_left carotid_ | 1.61±0.46 (1.22;1.99) | 2.02±0.74 (1.41;2.64) | 0.195 |
|  | TBR_right carotid_ | 1.61±0.41 (1.27;1.96) | 1.84±0.55 (1.38;2.30) | 0.369 |
|  | TBR_arch_ | 1.81±0.34 (1.52;2.10) | 2.20±0.58 (1.71;2.68) | 0.126 |
|  | TBR_descending_ | 1.94±0.39 (1.62;2.27) | 2.60±1.43 (1.40;3.80) | 0.231 |
|  | TBR_ascending_ | 2.10±0.89 (1.36;2.85) | 2.26±0.74 (1.64;2.87) | 0.713 |
|  | TBR_abdominal_ | 1.92±0.45 (1.54;2.29) | 3.82±1.63 (2.45;5.18) | 0.007 |
|  | TBRleft_iliac | 1.66±0.22 (1.43;1.81) | 2.49±0.98 (1.67;3.31) | 0.049 |
|  | TBRright_iliac | 1.86±0.39 (1.53;2.19) | 2.56±1.13 (1.62;3.50) | 0.118 |
| High Risk (n=8) | TBR_mean_ | 2.01±0.40 (1.67;2.88) | 3.70±1.24 (2.58;5.69) | 0.003 |
|  | TBR_left carotid_ | 1.73±0.49 (1.31;2.14) | 2.71±1.60 (1.37;4.05) | 0.119 |
|  | TBR _right carotid_ | 1.76±0.56 (1.29;2.22) | 2.97±1.67 (1.57;4.38) | 0.071 |
|  | TBR_arch_ | 2.01±0.30 (1.76;2.27) | 3.53±2.51 (1.44;5.63) | 0.111 |
|  | TBR_descending_ | 1.98±0.42 (1.64;2.33) | 3.63±1.19 (2.63;4.63) | 0.002 |
|  | TBR_ascending_ | 2.08±0.38 (1.76;2.40) | 3.07±0.84 (2.38;3.77) | 0.008 |
|  | TBR_abdominal_ | 2.57±0.91 (1.81;3.32) | 6.17±1.85 (4.62;7.71) | 0.001 |
|  | TBRleft_iliac | 1.95±0.32 (1.68;2.21) | 3.78±1.29 (2.70;4.86) | 0.002 |
|  | TBRright_iliac | 2.07±0.55 (1.61;2.52) | 3.71±1.37 (2.56;4.86) | 0.007 |

**Table 3**:

Intraclass correlation coefficients, along with 95% confidence for inter- and intra reader.

|  | **ICC values using the maximum TBR Measurement (^18^F/^68^Ga)** | | | | | | | |
| --- | --- | --- | --- | --- | --- | --- | --- | --- |
| Parameter | Left carotid | Right carotid | Aortic arch | Descending | Ascending | Abdominal | Left iliac | Right iliac |
|
| Inter-reader | 0.92 | 0.93 | 0.97 | 0.9 | 0.95 | 0.94 | 0.92 | 0.89 |
| Agreement  (^18^F) | (0.90,0.99) | (0.83,0.98) | (0.95,0.99) | (0.74,0.96) | (0.94,0.97) | (0.90,0.97) | (0.84,0.97) | (0.87,0.93) |
| Inter-reader | 0.94 | 0.96 | 0.98 | 0.95 | 0.96 | 0.98 | 0.91 | 0.91 |
| Agreement  (^68^Ga) | (0.88,0.99) | (0.91,0.99) | (0.97,0.99) | (0.90,0.97) | (0.93,0.98) | (0.97,0.99) | (0.86,0.95) | (0.80,0.97) |
| Intra-reader | 0.9 | 0.82 | 0.95 | 0.84 | 0.89 | 0.97 | 0.88 | 0.82 |
| Agreement  (^18^F) | (0.70,0.97) | (0.60,0.94) | (0.86,0.98) | (0.66,0.95) | (0.69,0.96) | (0.92,0.99) | (0.67,0.96) | (0.58,0.93) |
| Intra-reader | 0.81 | 0.89 | 0.96 | 0.97 | 0.85 | 0.9 | 0.87 | 0.89 |
| Agreement  (^68^Ga) | (0.51,0.91) | (0.68,0.97) | (0.87,0.99) | (0.91,0.99) | (0.68,0.95) | (0.70,0.97) | (0.63,0.96) | (0.71,0.96) |
| Data in parentheses are 95% confidence intervals | | | | | | | | |

**Table 4**:

Graphs of correlation between uptake of each arterial segment and risk factors attached

| **Correlations (^18^F-FDG)** | | | | | | | | | | |
| --- | --- | --- | --- | --- | --- | --- | --- | --- | --- | --- |
|  | PB | Hyperchol | HTN | Smoker | DM | Fam_Hist | Prior_Event | Age | Gender | BMI |
| Left_Carotid | NS | NS | NS | NS | NS | NS | NS | .582^*^ | NS | .369 |
| Right_Carotid | NS | NS | NS | NS | NS | NS | NS | .547^*^ | NS | NS |
| Arch | NS | NS | NS | NS | NS | NS | NS | NS | NS | NS |
| Descending | NS | NS | NS | NS | NS | NS | NS | .526^*^ | NS | .502^*^ |
| Ascending | NS | NS | NS | NS | NS | NS | NS | NS | NS | NS |
| Abdominal | NS | NS | .527^*^ | NS | .505^*^ | NS | NS | NS | NS | .600^*^ |
| Left_iliac | NS | NS | .NS | NS | NS | NS | NS | NS | NS | NS |
| Right_iliac | NS | NS | NS | NS | NS | NS | NS | NS | NS | NS |
| Mean | NS | NS | .578^*^ | NS | NS | NS | NS | NS | NS | NS |
| PB | NS | .515^*^ | NS | .552^*^ | NS | NS | 629^**^ | NS | NS | NS |
| **. Correlation is significant at the 0.01 level (2-tailed). | | | | | | | | | | |
| *. Correlation is significant at the 0.05 level (2-tailed). | | | | | | | | | | |
| PB:Plaque burden; HTN: Hypertension; Hyperchol: Hypercholesterolemia; | | | | | | | | | | |
| CAD: Cardiovascular disease; BMI: Body mass index. | | | | | | | | | | |

| **Correlations (^68^Ga-DOTATATE)** | | | | | | | | | | |
| --- | --- | --- | --- | --- | --- | --- | --- | --- | --- | --- |
|  | PB | Hyperchol | HTN | Smoker | DM | Fam_Hist | Prior_Event | Age | Gender | BMI |
| Left_Carotid | NS | NS | NS | NS | NS | NS | NS | NS | NS | NS |
| Right_Carotid | NS | NS | NS | NS | NS | NS | NS | NS | NS | NS |
| Arch | .575^*^ | NS | NS | NS | NS | NS | .582^*^ | .521^*^ | NS | NS |
| Descending | NS | NS | .571^*^ | NS | NS | NS | NS | .510^*^ | NS | NS |
| Ascending | NS | NS | NS | NS | .562^*^ | NS | NS | NS | NS | NS |
| Abdominal | .545^*^ | NS | .653^**^ | NS | NS | NS | NS | NS | NS | .558^*^ |
| Left_iliac | NS | NS | NS^*^ | NS | NS | NS | NS | .583^*^ | NS | .651^**^ |
| Right_iliac | NS | NS | NS^*^ | NS | NS | NS | NS | NS | NS | .624^**^ |
| Mean | .517^*^ | NS | .600^*^ | NS | NS | NS | NS | .558^*^ | NS | NS |
| PB | NS | .515^*^ | NS | .552^*^ | NS | NS | .629^**^ | NS | NS | NS |
| **. Correlation is significant at the 0.01 level (2-tailed). | | | | | | | | | | |
| *. Correlation is significant at the 0.05 level (2-tailed). | | | | | | | | | | |
| PB:Plaque burden; HTN: Hypertension; Hyperchol: Hypercholesterolemia; | | | | | | | | | | |
| CAD: Cardiovascular disease; BMI: Body mass index. | | | | | | | | | | |

**Table 5**

Comparison of focal ^68^Ga-DOTATATE and ^18^F-FDG uptake in eight arterial segements

| Artery | Tracer | Mean±SD | 95%CI for mean | P value* |
| --- | --- | --- | --- | --- |
| Left carotid | ^18^F-FDG | 1.67±0.46 | 1.42,1.91 | ＜0.05 |
|  | ^68^Ga-DOTATATE | 2.37±1.26 | 1.70,3.04 |  |
| Right carotid | ^18^F-FDG | 1.68±0.48 | 1.43,1.94 | ＜0.05 |
|  | ^68^Ga-DOTATATE | 2.41±1.34 | 1.69,3.12 |  |
| Aortic arch | ^18^F-FDG | 1.91±0.33 | 1.73,2.09 | NS |
|  | ^68^Ga-DOTATATE | 2.86±1.89 | 1.86,3.87 |  |
| Descending  aorta | ^18^F-FDG | 1.96±0.39 | 1.76,2.17 | ＜0.01 |
|  | ^68^Ga-DOTATATE | 3.12±1.38 | 2.38,3.85 |  |
| Ascending  aorta | ^18^F-FDG | 2.09±0.66 | 1.74,2.44 | ＜0.05 |
|  | ^68^Ga-DOTATATE | 2.67±0.87 | 2.20,3.13 |  |
| Abdominal  aorta | ^18^F-FDG | 2.24±0.77 | 1.83,2.65 | ＜0.01 |
|  | ^68^Ga-DOTATATE | 4.99±2.07 | 3.89,6.10 |  |
| Left iliac  artery | ^18^F-FDG | 1.62±0.31 | 1.62,1.95 | ＜0.01 |
|  | ^68^Ga-DOTATATE | 3.13±1.26 | 2.44,3.82 |  |
| Right iliac  artery | ^18^F-FDG | 1.96±0.47 | 1.71,2.21 | ＜0.01 |
|  | ^68^Ga-DOTATATE | 3.14±1.35 | 2.42,3.86 |  |
| Mean | ^18^F-FDG | 1.91±0.40 | 1.70,2.13 | ＜0.01 |
|  | ^68^Ga-DOTATATE | 3.08±1.21 | 2.44,3.73 |  |
| *p≤0.05 is considered significant. CI = Confidence interval | | | |  |

**Table 6**

Risk factors of patients

| **Patient NO.** | **Risk facors (Y/N)** | | | | | | | | | |
| --- | --- | --- | --- | --- | --- | --- | --- | --- | --- | --- |
|  | **PB** | **Hyperchol** | **HTN** | **Smoker** | **DM** | **Fam Hist. of CVD** | **CVD** | **Age** | **Gender** | **BMI** |
| 1 | 13 | Y | Y | Y | Y | N | N | 73 | M | 38.06 |
| 2 | 4 | N | Y | N | N | N | N | 63 | M | 19.82 |
| 3 | 8 | N | Y | N | N | N | N | 66 | M | 26.59 |
| 4 | 1 | N | N | Y | N | N | N | 48 | M | 22.15 |
| 5 | 0 | N | N | N | N | Y | N | 56 | M | 28.6 |
| 6 | 7 | N | Y | N | Y | Y | Y | 77 | M | 24.86 |
| 7 | 11 | N | N | N | N | Y | N | 58 | M | 21.16 |
| 8 | 12 | N | Y | Y | N | Y | N | 64 | M | 24.76 |
| 9 | 1 | N | Y | N | N | N | N | 51 | F | 20.57 |
| 10 | 12 | Y | Y | Y | Y | N | N | 52 | M | 28.36 |
| 11 | 0 | N | N | N | N | N | N | 71 | F | 20.08 |
| 12 | 25 | N | Y | Y | N | N | Y | 61 | M | 24.22 |
| 13 | 27 | Y | Y | Y | N | N | Y | 74 | F | 20.06 |
| 14 | 12 | Y | Y | N | N | N | N | 72 | M | 34.14 |
| 15 | 5 | N | Y | N | Y | N | N | 63 | M | 26.58 |
| 16 | 13 | Y | N | N | N | N | N | 67 | F | 16 |
| PB:Plaque Burden; HTN: Hypertention; DM: Diabetes; CVD: Cardiovascular Disease; BMI: Body Mass Index | | | | | | | | | | |
